# Removal of Copper(II) in the Presence of Sodium Dodecylobenzene Sulfonate from Acidic Effluents Using Adsorption on Ion Exchangers and Micellar-Enhanced Ultrafiltration Methods

**DOI:** 10.3390/molecules27082430

**Published:** 2022-04-09

**Authors:** Anna Wołowicz, Katarzyna Staszak, Zbigniew Hubicki

**Affiliations:** 1 Department of Inorganic Chemistry, Faculty of Chemistry, Institute of Chemical Sciences, Maria Curie-Sklodowska University, Maria Curie-Sklodowska Square 2, 20-031 Lublin, Poland; zbigniew.hubicki@mail.umcs.pl; 2 Institute of Chemical Technology and Engineering, Poznan University of Technology, Berdychowo St. 4, 60-965 Poznan, Poland; katarzyna.staszak@put.poznan.pl

**Keywords:** copper, sodium dodecylobenzene sulfonate, adsorption, removal, micellar-enhanced ultrafiltration

## Abstract

The selective removal of Cu(II) in the presence of sodium dodecylobenzene sulfonate from acidic effluents was made using the adsorption and micellar-enhanced ultrafiltration methods. Lewatit MonoPlus TP220 showed the best adsorption behavior in the systems containing Cu(II) in the presence of ABSNa50 surfactant compared to the other adsorbents (removal efficiency ≈ 100%, sorption capacity ≈ 10 mg/g). The kinetics followed the pseudo-second order kinetic equation. The Langmuir adsorption capacities were 110 mg/g (the system with ABSNa50 above CMC) and 130.38 mg/g (the system with ABSNa50 below CMC). The working ion exchange capacities were *C_w_* = 0.0216 g/mL and *C_w_* = 0.0135 g/mL. The copper removal by the micellar-enhanced ultrafiltration method was 76.46% (0.1 mol/L HCl).

## 1. Introduction

Heavy metals enter the environment through natural processes and through human activities. The rapid development of industries such as metal cleaning, tanning, battery making, plating, refining, pickling, etc., results in the discharge of millions of tons of heavy metal-containing wastewaters into the environment [1,2]. Nowadays, heavy metals are becoming one of the most hazardous environmental issues; therefore, such metals should be removed from the wastewater, in accordance with strict regulations, before they are directly or indirectly discharged into rivers, lakes, streams, etc. As reported in the literature, among the numerous methods used for heavy metal ion removal, ion exchange, adsorption, and membrane filtration are the most frequently investigated and applied [2,3]. Currently, adsorption is perceived as the most efficient and selective, as well as economic, method for heavy metal ions containing wastewater treatment due to its flexibility and simplicity of design, ease of operation, and insensitivity to toxic pollutant removal [4,5]. On the other hand, membrane processes are becoming more and more widespread due to their low costs and the possibility of the modernization of existing plants by adding membranes and due to the possibility of obtaining high efficiency removal, at about 99% [6]. Moreover, the membrane filtration process could be carried out at room temperature without reduction in yield efficiency.

Copper is considered to be the most valuable and commonly used metal in many industrial applications (strategic metal). Copper and their compounds are widely used in the electroplating process and in textile dyeing and as pigments for ceramics, glass, metal finishing, plastics, and etching. Depending on the application area, copper is also used with surfactants acting as wetting, emulsifying, or degreasing agents in, for example, the production of textiles (consuming about 10% of the world surfactant production), fibers, polymers, pharmaceuticals, food, oil recovery, and paints [7]. The wastewaters originating from the above mentioned branches of industry are rich in Cu(II) or/and surfactants, and their treatment can be troublesome. Copper, as well as some surfactants, exhibit high acute and chronic toxicity to aquatic organisms. Moreover, copper can cause serious kidney and liver damage in the human body [7,8]. On the other hand, surfactants are responsible for the formation of foams in effluent treatment plants and rivers and deteriorate the quality of the water [9]. Due to the above mentioned facts, the removal of Cu(II) and surfactant containing wastewaters using the micellar-enhanced ultrafiltration process (MEUF) as well as adsorption is very important, not only because of their toxicity but also because of the biodegradation metabolites of surfactants, which are more persistent. As the literature reports, both adsorption and MEUF were applied for the removal of copper ions from wastewaters with high efficiency, e.g., Cu(II) removal from synthetic wastewater in the presence of ligands such as citric, ethylenediamine-tetraacetic acid, and nitrilotriacetic acids (the MEUF method and the application of cetylpiridynium surfactants (cationic one) allows the obtaining of higher Cu(II) removal efficiency than in the case of sodium dodecylsulfate (SDS) (anionic one)) [10]; Cu(II), Pb(II), Fe(II), and Mn(II) removal from synthetic and real wastewaters mine drainage water with Cu(II) (polymer-enhanced ultrafiltration process (PEUF) with carboxylmethyl cellulose as a complexation agent) [11]; and Cu(II), Ni(II), and Cr(III) removal from synthetic wastewaters (PEUF, carboxylmethyl cellulose as a complexation agent, metal rejection efficiency > 98%) [12]. The removal of Cs(I), Sr(II), Mn(II), Co(II), Cu(II), Zn(II), and Cr(III) ions from aqueous solutions using the anionic SDS surfactant was investigated by Juang et al. [13]. The MEUF method was also applied for the removal of Cd(II), Pb(II), Cu(II), Ni(II), and Zn(II) using natural surfactants, such as deoxycholic acid and lecithins [14], as well as for the removal of Cu(II) and other heavy metals from real industrial wastewaters originating from a metal finishing plant, using the sodium salt of deoxycholic acid (DCA) [15]. The derivatives of cholesterol (DCA and taurocholic acid) and SDS applied in the MEUF method for removal of Cd(II), Cu(II), Ni(II), Pb(II) and Zn(II) show the strongest binding of SDS and DCA in single- and multicomponent solutions, respectively [16]. The removal of Cu(II) and other heavy metal ions from aqueous solutions by MEUF was also widely presented in [17,18,19], whereas the removal of Cu(II) using the adsorption method and different types of adsorbents was presented in, among others, the review papers, e.g., [8,20,21].

The objective of this research is to develop an efficient method for Cu(II) removal using the adsorption and MEUF methods in the presence of sodium dodecylobenzene sulfonate to simulate real industrial wastewater containing surfactants. A few sorbents, especially ion exchangers, were determined, and the best one was selected for further studies. Moreover, the adsorption capacities of the ion exchangers were determined by the static and column methods from the solutions containing anionic surfactant of a concentration below (↓CMC) and above (↑CMC) critical micelle concentration (CMC). Kinetic and equilibrium studies were presented. The adsorption process was monitored by scanning electron microscope (SEM) and atomic force microscope (AFM). In addition, the novelty of the work involved the use of the optical profilometer method to describe the adsorption process.

## 2. Results

### 2.1. Characterization of Adsorbents

Lewatit: MonoPlus TP220 (TP220), MonoPlus SR7 (SR7), or AF5 (AF5); Purolite: A400TL (A400TL), A830 (A830), or S984 (S984); and Dowex: PSR2 (PSR2) or PSR3 (PSR3) were applied in the removal of Cu(II) and their characteristics are depicted in Table 1.

These adsorbents belong to different types: chelating (TP220, S984), strongly (SR7, A400TL, PSR2, PSR3) and weakly basic (A830, TP220) ion exchangers, as well as the adsorbent without functional groups (AF5). The elementary analysis of the applied sorbents (0.2–200 mg) means that the percentage contents of carbon, hydrogen, nitrogen, and sulphur (%CHNS) were obtained (Table 2).

All the samples were measured four times, and the average contents of C, H, N, and S were calculated. On the basis of these results, the carbonaceous matrix of AF5 was confirmed. This adsorbent is composed of a large content of carbon (>93%), whereas the contents of the other elements were in the range from 0.02% to 1.58%. The other adsorbents are less abundant in carbon compared to Lewatit AF5 because they possess a different matrix, but %C is still high (93.81% (AF5) > 73.89% (PSR3) > 71.38% (PSR2) > 70.79% (SR7) ≈ 70.58% (TP220) > 59.64% (A400TL) > 46.95% (S984) ≈ 46.00% (A830)). The cross-linked polystyrenic ion exchange resins (TP220, SR7, PSR2, PSR3) possess a larger carbon content (close to 70–74%) compared to the polyacrylic ones (A400TL, A830, S984) (close to 46–60%). The content of %N is a result of the functional groups type. The largest content of nitrogen was found for the chelating or weakly basic ion exchange resins containing the polyamine functional groups (18.13% (S984) > 16.35% (A830) > 9.26% (TP220)). The content of nitrogen for the other adsorbents is much smaller and close to 4.5% or 2%. The nitrogen content is smaller by 75%–85% compared to that of S984. The percentage content of H and S is small for all the adsorbents examined (%H = 1.58%–0.89%; %S < 1.28%). The previous elementary analysis of C, N, and H contents for TP220 and Dowex M4195 (M4195) of similar bis-picolylamine functional groups shows the following composition of sorbents: %N = 10.86%, %C = 74.3%, %H = 7.06% (TP220) and %N = 9.43%; %C = 80.24%; %H = 6.95% (M4195) [22]. Kołodyńska et al. [23] prove that TP220 possesses 60.8%C, 8.6%N, 6.4%H, and 6.8%S, whereas M4195 has 68.5%C, 7.9%N, 6.5%H, and 3.4%S. The content of S is slightly higher than that obtained by us as a result of the application of other equipment, a different portion of the ion exchanger, and the difference in the pretreatment procedure before being used (the purification process was probably not satisfactory in this case). A higher S content in the case of TP220 could also be the result of the presence of sulfonic acid functional groups as a consequence of the sulfonation process of the ion exchange resin to improve the kinetic of the resin exchange. Additionally, M4195 is provided as a weak base or a partial H_2_SO_4_ salt (the resin is fully swollen and ready for the removal of metal ions when conjugated with the sulfuric acid salt). Another production process also influences the CHNS content. TP220 has been commercially available since 2007 as a product of the Lanxess company, whose production is based on Gabriel phthalimide synthesis. TP220 is obtained in a nitrogen atmosphere by the N-alkylation of poly(aminomethylstyrene) cross-linked with well-dried 2-chloroalkylpyridinein dioxane in the presence of triethylamine or anhydrous potassium carbonate, while M4195 is obtained by the reaction of bis[(2-pyridylmethyl)]amine with the chloromethylated styrene-divinylbenzene copolymer (produced in the early 1970s) [23]. Our previous studies showed that a similar content of CHN elements was obtained in the case of SR7 (%C = 64.9%, %H = 9.94%, and %N = 3%) [24] and A830 (%C = 43.28%, %H = 8.41%, and %N = 16.88%) [25].

The properties of porous materials depend on porosity, pore size, pore shape, pore wall thickness and shape, surface roughness, and surface area. The porous characteristics determine the properties of adsorbents and their applicability in the case of adsorption. The porous parameters, such as BET surface area (*S_BET_*), average pore size, and the volume [26,27] of the adsorbent applied for Cu(II) removal, were obtained and are presented in Table 2. The *S_BET_* is the largest in the case of AF5 being close to 1000 m^2^/g (the values are a little smaller than those provided by the product data sheets—1200 m^2^/g [28]). Due to the fact that this adsorbent is heterodispersive and the bead sizes are from 0.4 to 0.8 mm, the obtained *S_BET_* could be slightly different and depends on the fraction of beads taken into account (similar to other porous parameters). AF5 is composed of carbonaceous materials and is a different type of adsorbent compared to the other ion exchangers, whose *S_BET_* is usually much smaller, e.g., M4195 (bis-picolylamine functional groups) possesses the *S_BET_* = 7.1 m^2^/g [23], while for Purolite S957 (phosphonic and sulphonic functional groups) *S_BET_* = 14.9 m^2^/g [29]. The largest *S_BET_* was observed in the case of the monodyspersive Lewatit ion exchanger (*S_BET_* = 21.38 m^2^/g for TP220 > 19.69 m^2^/g for SR7).

The obtained average pore size corresponds well to the structure of the adsorbents. Macroporous adsorbents usually possess an average pore size larger than that of the microporous ones. The smallest average pore size was observed in the case of AF5 (2.31 nm) and for the microporous ion exchangers such as A400TL (2.35 nm) and PSR2 (10.08 nm). Among the macroporous ion exchangers, PSR3 possesses the smallest average pore size, whereas S984 has the largest one. For the adsorbents under discussion, at the point of zero charge the *pH_pzc_* was equal to 2.28 (TP220), 9.01 (S984), 1.48 (SR7), 1.03 (PSR3), 1.00 (PSR2), 1.00 (A400TL), 6.05 (A830), and 7.09 (AF5) [30] (obtained using the solid addition method [31]).

### 2.2. Kinetic Studies of Cu(II) Adsorption

The adsorption of Cu(II) by the ion exchangers under discussion from the systems of different acid concentrations (0.1–6 mol/L HCl–100 mg Cu(II)/L; 0.1–0.9 mol/L HCl–0.9–0.1 mol/L HNO_3_—100 mg Cu(II)/L) was discussed in the previous paper [30]. It was observed that among the eight adsorbents discussed, the best adsorption behavior towards Cu(II) was shown by TP220. In this paper, the adsorption of Cu(II) from the acidic solutions in the presence of ABSNa50 surfactant is discussed. The critical micelle concentration values obtained for the systems, 0.1–6 mol/L HCl–100 mg Cu(II)/L–ABSNa50, were presented previously (system, CMC vales; water–100 mg Cu(II)/L−ABSNa50,0.18 mmol/L; 0.1 mol/L−100 mg Cu(II)/L−ABSNa50,0.15 mmol/L; 1 mol/L−100 mg Cu(II)/L−ABSNa50,0.09 mmol/L; 3 mol/L−100 mg Cu(II)/L−ABSNa50,0.05 mmol/L; 6 mol/L−100 mg Cu(II)/L−ABSNa50,0.07 mmol/L) [32]. The kinetic curves were obtained based on the static studies described in Section 3.3, which were carried out at the phase contact time in the range of 1 min to 24 h. The sorption capacity, (*q_e_*) (mg/g), the amount of Cu(II) ions adsorbed after time *t*, (*q_t_*) (mg/g), and the percentage removal, (%*R*), were calculated using the following formulae [33]:

– sorption capacity (*q_e_*) (mg/g):
  (1) $$q_{e}=\frac{(C_{0}-C_{e})\times V}{m}$$
– the amount of Cu(II) ions adsorbed after time *t* (*q_t_*) per weight unit of sorbent under non-equilibrium conditions:
  (2) $$q_{t}=\frac{(C_{0}-C_{t})\times V}{m}$$
– percentage removal (%*R*):
  (3) $$\%R=\frac{C_{0}-C_{t}}{C_{0}}\times 100\%$$
  where *C*_0_ (mg/L)—the initial concentration of Cu(II) in the water phase, *C_e_* (mg/L)—the equilibrium concentrations of Cu(II) in the water phase, *C_t_* (mg/L)—the concentration of Cu(II) in the water phase after *t*, *V* (L)—the volume of solution, and *m* (g)—the mass of dry sorbent.

**Figure 1:**
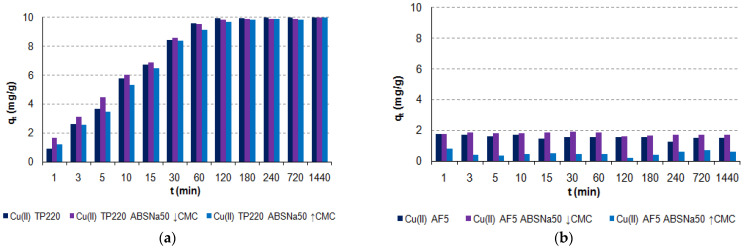

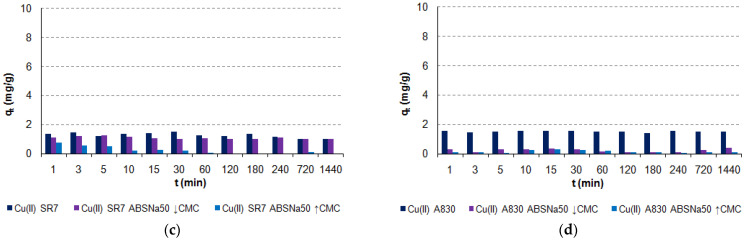
Effect of phase contact time on the removal of Cu(II) ions by: (**a**) TP220; (**b**) AF5; (**c**) SR7; and (**d**) A830 (*m* = 0.5 ± 0.0005 g, *V* = 50 mL, *C*_0_ = 100 mg/L, *A* = 8, *t* = 1–1440 min, *T* = 295 K).

The adsorption of Cu(II) during 1 min–24 h is shown in Figure 1.

As follows from the phase contact time experiments and the kinetic curves, TP220 shows the best adsorption behavior in the systems containing Cu(II) in the presence of ABSNa50 surfactant compared to the other ones. The presence of ABSNa50 ↓CMC as well as ↑CMC caused in most cases (for the anion exchangers, e.g., SR7, A830) the reduction in the adsorption capacities, which was the greatest when the surfactant concentration was higher (↑CMC). Moreover, for these anion exchangers the adsorption capacities were small in both the systems without ABSNa50 and in its presence. The adsorption capacities did not exceed 2 mg/g (systems without ABSNa50), 1.2 mg/g (systems with ABSNa50 ↓CMC), or 0.8 mg/g (systems with ABSNa50 ↑CMC). However, for AF5 (adsorbent without ion exchange functional groups), the presence of ABSNa50 ↓CMC caused a slight increase in the adsorption capacities compared to the systems without the surfactant. When the concentration of ABSNa50 is ↑CMC, the reduction in adsorption capacities is observed. Taking into account the results obtained for TP220, it can be observed that this ion exchanger shows the greatest adsorption ability towards Cu(II) from the acidic solutions without the surfactant [30], as well as in a more complicated system with the anionic surfactant (this paper). At the beginning of the adsorption process (*t* = 1–30 min), when the concentration of ABSNa50 is ↓CMC, a slightly greater adsorption capacity is observed. For *t* > 30 min the adsorption capacities are slightly smaller. Moreover, in the systems with the ABSNa50 ↑CMC, the reduction in adsorption capacities is observed, but it does not exceed 8%.

The presented system is complicated because it contains many components, such as the adsorbent (with or without functional groups), Cu(II), strong electrolyte HCl, as well as the anionic surfactant. Similar complex systems exist in real solutions. The different behavior of the adsorbents towards Cu(II) sorption in the presence of ABSNa50 is affected by many factors, e.g., the various properties of the adsorbents (operating ion exchange capacity, physical strength, and resistance to fouling by high molecular weight organic anions) and the solution composition, as well as the concentration and properties of the surfactant. Moreover, the Cu(II) removal efficiency is related to the presence of functional groups, which results in the ion exchange capacities of adsorbents [34,35].

The literature data indicate that the adsorption of surfactants on adsorbents occurs by different mechanisms, such as hydrogen binding, electrostatic interactions, π-π interactions, and ion pairing, as well as Van der Waals forces [36]. On the other hand, copper can be bound to the ion exchangers by adsorption, ion-exchange, and chelation, as well as mixed mechanism [20,28]. In the presented solutions, copper ions in the HCl solutions can exist in different forms depending on the hydrochloric HCl concentration as well as the total concentration of the chloride ions [20,28]. The speciation of Cu(II) as a function of log from the total chloride concentration (100 mg Cu(II)/L) was presented in the previously published paper [20]. Moreover, the copper species in the HCl solutions were described in the following papers: [30,37,38]. The copper ions exist as Cu^2+^, CuCl^+^, CuCl_2_, CuCl_3_^−^, and CuCl_4_^2−^ in the examined systems, and the forms of Cu species change their charge from positive, through neutral, and then to negative [22,30,37,38,39]. The applied anion exchangers possess a different matrix, such as cross-linked polystyrene, e.g., TP220, SR7, and cross-linked polyacrylic, e.g., A830 or the carbonaceous ones, e.g., AF5. As was confirmed, the anion exchange resins of the polyacrylic matrix are more hydrophilic in nature compared to the polystyrenic ones. Due to this fact, they should demonstrate more beneficial exchange equilibria and kinetics towards large organic ions such as surfactant, which in turn means a weaker van der Waals type attraction: the resin matrix—the hydrocarbon structure of an organic counter-ion (non-ionic moieties) [34,40]. Additionally, under acidic conditions a significant uptake of organic compounds can occur [41]. Moreover, on the anion exchange resins not only does the ordinary anion exchange with the participation of the ionogenic groups take place but these resins also have the potential for hydrophobic interactions between the surfactant anion and the polymeric matrix of adsorbents when the surfactant anion also contains a hydrophobic centre. As is known, ABSNa50 (the sodium salt of alkyl benzene sulfonic acid) belongs to the anionic surfactants group and contains two parts: the anionic center (hydrophilic head) and the hydrophobic tail (the hydrocarbon chain); therefore, in this system, the mixed-mode type of interactions with anion exchangers that possess both anion-exchange ionogenic groups and a matrix take place, and the interaction with the hydrophobic part of surfactant takes place [35,40,42,43]. Additionally, the π-π interactions (hydrophobic interactions, “like attract like”) between the aromatic ring of the ABSNa50 surfactant and the matrix rings of the adsorbents could proceed.

**Figure 2:**
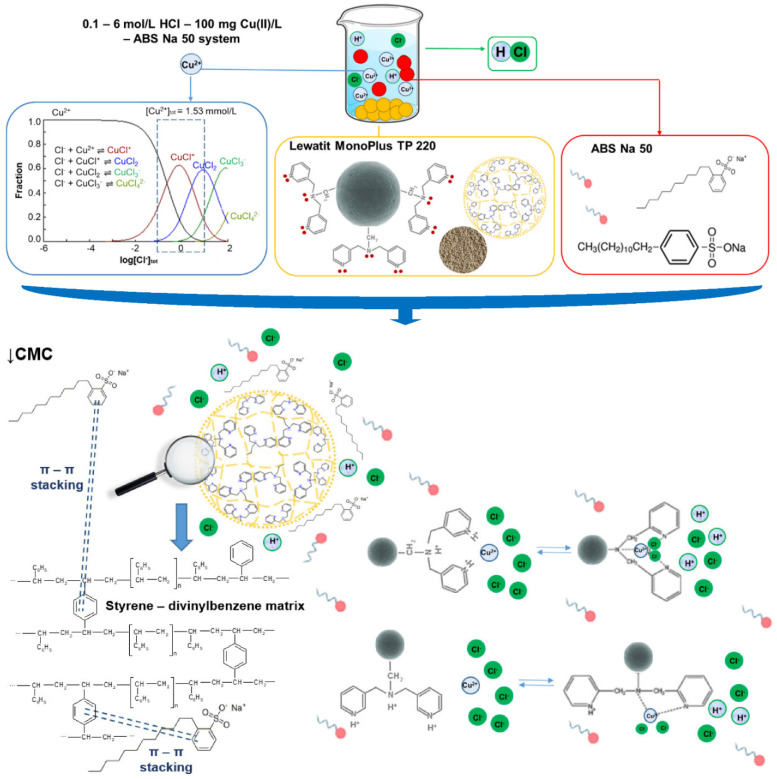

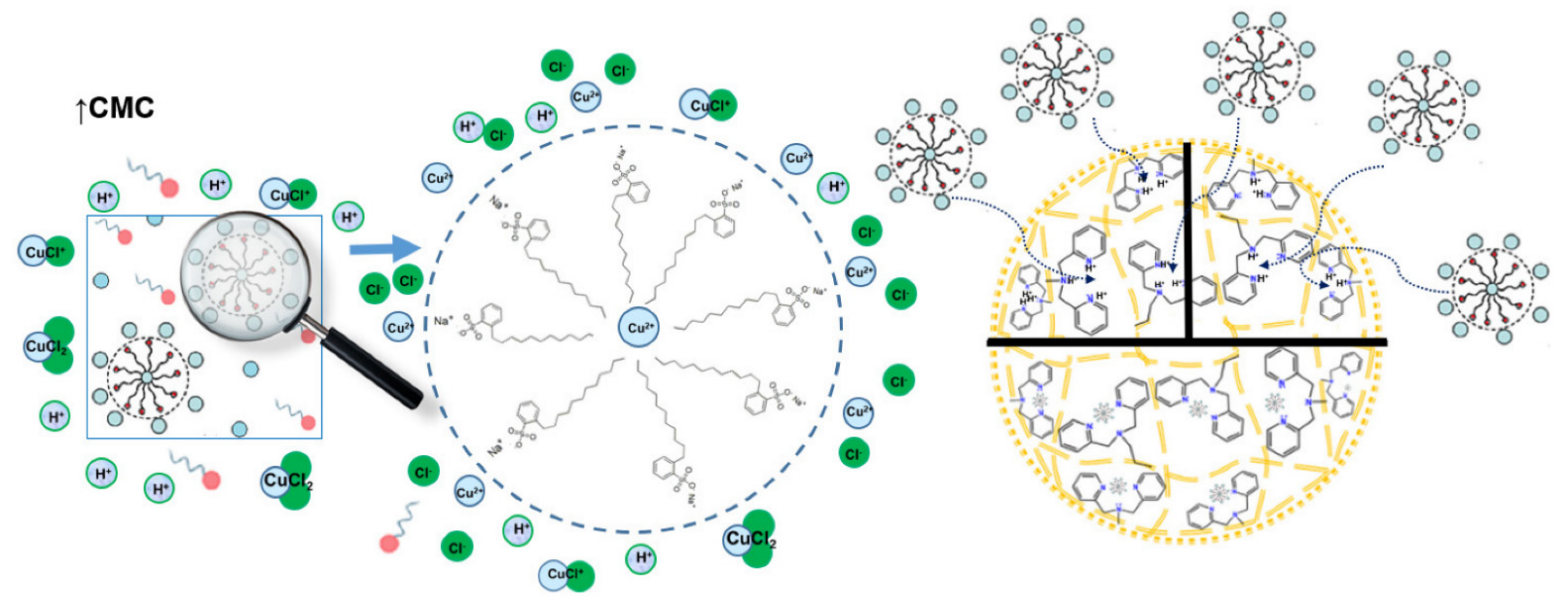
The mechanism of Cu(II) adsorption in the presence of ABSNa50.

Lewatit MonoPlus TP220 is a chelating resin composed of synthetic copolymers with covalently bound immobilized side chains which possess three nitrogen donor atoms (donors of free electron pairs); two of them are in the aromatic pyridyl groups, while one is in the tertiary amine functional group (two aromatic and one aliphatic) that acts as the polydentate ligand that is the three-dentate ligand [44]. The nitrogen atom of the pyridyl group is a stronger reducing agent than that of the tertiary amine functional group. Such nitrogen donor atoms (Lewis bases) can interact strongly with the toxic metal ions (Lewis acids) through the coordination-type interactions [45]. Copper is a Lewis acid, a borderline soft metal cation, and, due to its incomplete 3d orbital, it has coordination properties and can form inner-sphere complexes with the Lewis base or a ligand (electron pair donor) [46]. TP220 is also a polymer with weak-base functionalities, due to the presence of pyridine or tertiary amines functional groups; therefore, it can behave in the hydrochloric acid solutions as the weakly basic anion exchanger, and its functional groups are capable of protonation. The protonation of the bis(2-pyridylmethyl)amine functional groups of the TP220 ion exchanger is affected by the pH value. At low pH values, three nitrogen atoms of the bis(2-pyridylmethyl)amine functional groups are protonated; at medium pH only one is, while none of them is protonated at the pH values above 3.5. When the bis(2-pyridylmethyl)amine functional group is attached to the resin, the pyridyl nitrogen atoms are more readily deprotonated in the chloride than the sulphate media. In the chloride solutions, the aliphatic amine is much more deprotonated (the changes of dissociation are constant when the secondary amine is converted to the tertiary amine when it is attached to the matrix of the resin) [23,47]. At acidic pH, two pyridyl nitrogen donor atoms are available to form a bidentate complex with copper ions, and copper uptake occurs through the Lewis acid–base interactions [46,48]. The effect of the chloride ion concentration on the copper-adsorption ability was described previously in [49]. As was found [49], the copper adsorption shows slight sensitivity to pH, being unaffected by the degree of resin protonation. The Lewis interactions are strong enough to break up any chloro-complexes through the interactions between the metal species and the nitrogen donor atoms. In the HCl solutions containing copper ions and anionic surfactant, the electrostatic interactions between the functional groups and the negative charge copper species (when the HCl concentration is high), as well as with the anionic part of the surfactant, can take place when the surfactant concentration is low (↓CMC). Moreover, the hydrophobic characteristics of the solution components can impact significantly on the ion-exchange sorption of organic compounds. The hydrophobic domains on the organic compounds decrease the entropy of the solution, which results in a thermodynamically favorable removal of the compound from the solution. In the literature, this is called an entropy-assisted sorption. Screening effects, as well as the de-solvation of molecules, can also influence the adsorption uptake [40,42,43]. When the concentration of the anionic surfactant is ↑CMC, the micelles between the copper ions and the anionic surfactant are formed. Based on the particle size analysis (Zetasizer Nano ZS Malvern), the diameter of the ABSNa50 surfactant molecules in the 0.1 mol/L HCl solution was 4.427 nm, and the polydispersity index (PdI) was 0.335. After introduction of Cu(II) into the solution (↑CMC), the diameter of the micelles was 5.98 nm, while the PdI decreased and was equal to 0.245. The possible mechanism of Cu(II) adsorption in the presence of ABSNa50 was proposed and is presented in Figure 2.

### 2.3. Kinetic and Isotherm Parameters of Adsorption System

For the design of an efficient and effective adsorption system, the kinetic and equilibrium data are required. The kinetic studies allow the estimation of the rate of contaminant elimination, whereas the adsorption isotherms to determine adsorbent effectiveness evaluation and applicability are by the maximum adsorption capacity calculation.

#### 2.3.1. Kinetic Parameters of Adsorption

**Figure 3:**
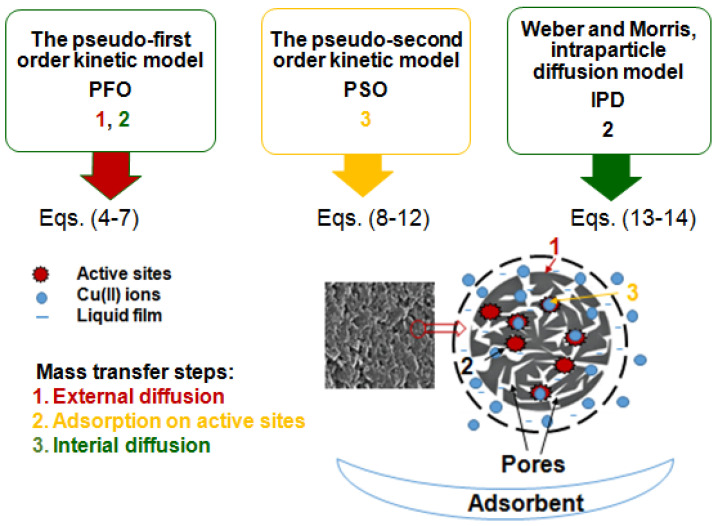
Kinetic models and error analysis summarizing.

Knowledge of adsorption kinetics allows for the entire adsorption process to be as effective as possible. The pseudo-second order kinetic equation (PSO), the pseudo-first order kinetic equation (PFO), the Lagergren equation, and the intraparticle diffusion (IPD) kinetic models are the most often used by researchers for adsorbate–adsorbent systems description. The kinetic models as well as the error analysis are presented below and in Figure 3.

**PFO:** non-linear equation (NL), linear equation (L), kinetic parameters (KP)
(4) $$\mathrm{NL}:\;\;\;\frac{dq_{t}}{dt}=k_{1}\left(q_{e}-q_{t}\right)$$
(5) $$L:\;\;\;\log\left(q_{e}-q_{t}\right)=\log q_{e}-\frac{k_{1}}{2.303}t$$
(6) $$\mathrm{KP}:\;\;\;k_{1}=-2.303\times slope$$
(7) $$q_{e}=10^{intercept}$$
**PSO:**
(8) $$\mathrm{NL}:\;\;\;\frac{dq_{t}}{dt}=k_{2}{\left(q_{e}-q_{t}\right)}^{2}$$
(9) $$L:\;\;\;\frac{t}{q_{t}}=\frac{1}{k_{2}q_{e}^{2}}+\frac{1}{q_{e}}t$$
(10) $$\mathrm{KP}:\;\;\;k_{2}=\frac{slope^{2}}{\mathrm{intercept}}$$
(11) $$q_{e}=\frac{1}{slope}$$
(12) $$h=k_{2}q_{e}^{2}$$
**IPD**:
(13) $$L:\;\;\;q_{t}=k_{i}t^{1/2}$$
(14) $$\mathrm{KP}:\;\;\;k_{i}=slope$$
**Error analysis:**
  - *MPSD*—the Marquardt’s percent standard deviation:
    (15) $$MPSD=\overset{n}{\underset{i=1}{\sum }}{\left(\frac{q_{e\;exp}-q_{e\;cal}}{q_{e\;exp}}\right)}_{i}^{2}$$
  - *R*^2^—the determination coefficient:
    (16) $$R^{2}=1-\frac{\sum {\left(q_{e\;exp}-q_{e\;cal}\right)}^{2}}{\sum {\left(q_{e\;exp}-q_{e\;mean}\right)}^{2}}$$
  - $R_{adj}^{2}$ the adjusted R-squared
    (17) $$R_{adj}^{2}=1-\left[\frac{\left(1-R^{2}\right)\left(n-1\right)}{n-k-1}\right]$$
    where *q_e_* and *q_t_* (mg/g)—the Cu(II) amounts sorbed at the equilibrium and at time t; *k*_1_ (1/min), *k*_2_ (g/mg min), *k_i_* (mg/g min^0.5^)—the rate constants of Cu(II) adsorption determined from the PFO, PSO, and IPD models; *h* (mg/g min)—the initial sorption rate; *q_e_*_,*exp*_ (mg/g)—the amount of Cu(II) adsorbed at equilibrium; *q_e_*_,*cal*_ (mg/g)—the calculated amount of Cu(II) adsorbed; *q_e,mean_* (mg/g)—measured by the means of the *q_e_*_,*exp*_ values; *k*—the number of independent regressors; and *n*—the points number.

**Figure 4:**
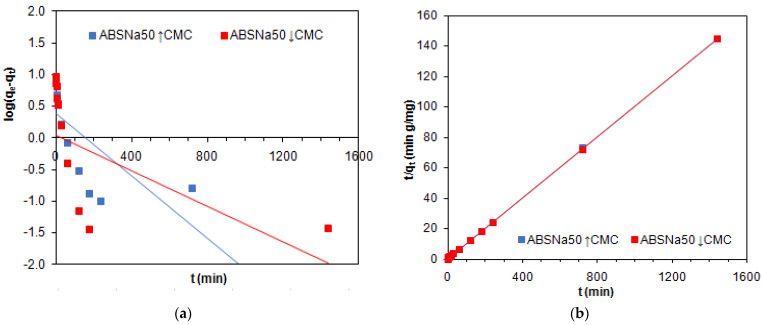

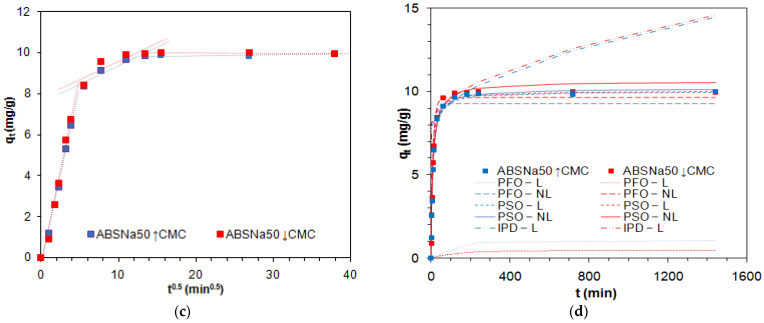
Plots obtained based on (**a**) PFO, (**b**) PSO, and (**c**) IPD models applied for the adsorption of Cu(II) in the presence of ABSNa50 on TP220 and (**d**) the linear (L) and non-linear (NL) fitting of PFO, PSO, IPD kinetic models to experimental data.

The PFO, PSO, and IPD equations were applied for the kinetic parameter determination. Taking into account the experimental points position on the kinetic curve and the adsorption dependence on time, the kinetic parameters were calculated only in the system with TP220. The kinetic parameters values were calculated based on the PFO, PSO, and IPD kinetic equations for TP220 (Table 3), and the appropriate figures are given in Figure 4 (PFO: log(*q_e_*−*q_t_*) vs. *t*—Figure 4a, PSO: *t*/*q_t_* vs. *t*—Figure 4b, IPD: *q_t_* vs. *t*^0.5^—Figure 4c), respectively.

In the case of the systems with SR7, AF5, A400TL, PSR2, PSR3, A830, and S984 adsorbents, the Cu(II) adsorption was not efficient enough, and the amount of Cu(II) adsorbed at time t (*q_t_*) was at a similar level for the entire time range (1–1440 min), making the calculations of the kinetic parameters impossible.

As was found, the PFO model (both the L and the NL regressions) did not find applicability in the description of Cu(II) adsorption from acidic systems containing ABSNa50 surfactant on TP220. The determination coefficients obtained based on the PFO-L model are small (*R*^2^ = 0.277 and 0.476), and the experimental values of the adsorption capacities (*q_e_*_,*exp*_ = 9.97 and 9.99 mg/g) are not in line with the calculated values (*q_e_*_,*cal*_ = 2.39 and 1.08 mg/g). Moreover, the graph *log(q_e_−q_t_)* vs. *t* was not linear (Figure 4a). Much higher values of the determination coefficient (*R*^2^ = 0.975 and 0.989) and greater compatibility of the experimental (*q_e_*_,*exp*_ = 9.97 and 9.99 mg/g) and obtained (*q_e_*_,*cal*_ = 9.25 and 9.63 mg/g) adsorption capacity values were found using the PFO-NL kinetic model, but the *R*^2^ values were not the highest compared with the *R*^2^ obtained based on the other kinetic models. Additionally, the *MPSD* values were small (PFO–NL: *MPSD* = 0.115 and 0.026) but much higher or similar compared to *MPSD* for PSO-NL (*MPSD* = 0.027).

The Weber and Morris model (IPD) was applied in three different forms as a plot *q_t_* plotted against *t*^1/2^ (the square root of time), as: (1) a straight line that is forced to pass through the origin; (2) a straight line that does not necessarily pass through the origin (intercept exists); and (3) a multi-linearity plot composed of two or three steps involving the whole adsorption process. According to (3), in the start step the external surface or instantaneous adsorption occurs (3a), followed by the gradual adsorption, in which the intraparticle diffusion is controlled (3b), and then, the last step is the final equilibrium, where the solute moves slowly from the larger pores to the micropores, resulting in a slow adsorption rate (3c). In the case of (2), when the straight line does not need to pass through the origin, the intercept exists and is proportional to the extent of the thickness of the boundary layer. Most of the intercepts reported in the literature have positive values. This fact indicates that the adsorption process occurs within a short period of time and is fast [50]. The graphs obtained based on the Weber–Morris equation for Cu(II) in the presence of ABSNa50 surfactant adsorption on TP220 (Figure 4c) illustrate multilinearity, and the adsorption data can be fitted with three straight lines. As was found, the straight line determined from the second part of the plot does not pass through the origin, indicating that not only the intraparticle diffusion affects the adsorption rate [51]. The values of the intraparticle diffusion rate constants *k_i_* were 0.18 mg/g min^0.5^ in both cases. In addition, the determination coefficients (*R*^2^ = 0.924 and 0.757) possess small values.

Based on the kinetic parameters given in Table 3 for the PSO model, both with the L and the NL regressions, it can be concluded that the PSO model gives the best fit to the experimental data. The applicability of the PSO-L model was proved not only by the high values of the determination coefficient (*R*^2^ = 1) but also by the values of the adsorption capacities (*q_e_*_,*exp*_ = 9.97 and 9.99 mg/g), which are very similar to those calculated (*q_e_*_,*exp*_ = 10.0 and 10.01 mg/g). For the PSO-NL model, the *MPSD* values are the smallest (*MPSD* = 0.027), while the *R^2^* and *R^2^_adj_* values are the largest (*R*^2^ = 0.996 and 0.991; *R*^2^*_adj_* = 0.995 and 0.989), indicating that this model found applicability in Cu(II) in the presence of ABSNa50 adsorption on TP220. The applicability of the PSO model with both the L and the NL regressions was also proved by the fitting plots, which are given in Figure 4d. As was found in the literature, the copper ion adsorption without surfactants followed the PSO model, e.g., the Cu(II) adsorption on the H_3_PO_4_-activated rubber wood sawdust (*S_BET_* = 1673.86 m^2^/g) (0.5 g; 100 mL; pH 6; *C*_0_ = 20 mg/L); the PSO model; %*R* = 80–90% (30 min); the equilibrium time = 240 min; the rate-limiting step: film diffusion (low concentrations) and particle diffusion (high concentration) [52]; the Cu(II) adsorption on Purolite S940 (*S_BET_* = 15.8 m^2^/g) and Purolite S950 (*S_BET_* = 15.7 m^2^/g) chelating ion exchangers (0.20 g, *C*_0_ = 20 mL of 0.001 M CuCl_2_, agitation speed 180 rpm, *T* = 298 K, time 2 h); the PSO model; the equilibrium time = 40 min (0.001 mol/L, all ion exchangers); 5 min (0.005 mol/L, Purolite A950) [53]; the Cu(II) adsorption on Purolite S930 (1 g/L *C*_0_ = 100, 300 mg/L, pH = 5, *T* = 293, 303 K, time 1 min–24 h); the PSO model; %*R* = 40% (4 h) [54]; the Cu(II) adsorption on Lewatit MonoPlus TP220 (0.5 g; 50 mL; *C*_0_ = 100 mg/L, agitation speed 180 rpm, amplitude 8, *T* = ambient, time 1 min–4 h); the PSO model; %*R* = 91–100% (depending on HCl, HNO_3_ concentration); the equilibrium time > 120 min (HCl), >60 min (HCl–HNO_3_ systems) [30], etc. The literature reports a few examples of Cu(II) adsorption in the presence of non-ionic surfactants [55,56], but the kinetics of the adsorption process in the systems containing heavy metal ions and surfactant is not described in detail. Cu(II) adsorption (0.5 g, 100 mL, *C*_0_ = 0.006 mol/L for OS-20 and CuCl_2_, 400 rev/min, 293 K), in the presence of oxyethylated alcohols (OS-20, non-ionic surfactant) on Purolite C 106 (weakly acidic cation exchange resin (WAC), forms H^+^, macroporous (macr.), polyacrylic matrix (PAc), functional groups (FG)—COOH) [55]; Cu(II) adsorption (0.5 g, 100 mL, *C*_0_ = 0.006 mol/L for ALM-10 and CuCl_2_, 400 rev/min, 293 K), in the presence of nonionic surfactant alkylmonoethers (ALM-10) on Duolite ES 468 (WAC), forms H^+^, macr., PAc, FG: -COOH) [56], indicating that the systems could be described by the intraparticle diffusion model.

#### 2.3.2. Isotherm Parameters of Adsorption

**Figure 5:**
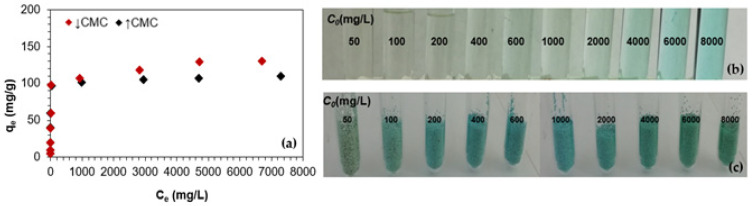
(**a**) Adsorption isotherm; (**b**) liquid and (**c**) ion exchanger phases after the copper adsorption in the presence of ABSNa50 surfactant on TP220.

The equilibrium studies, as well as the analysis of the experimental results of the adsorption isotherms, are important in developing the adsorption design on a larger scale. The adsorption isotherm plots for Cu(II) adsorption in the presence of ABSNa50 surfactant onto TP220, obtained by plotting the amount of Cu(II) ions adsorbed at equilibrium, and the adsorption capacities (*q_e_*) values versus the equilibrium concentration of the solute (*C_e_*) are depicted in Figure 5.

**Figure 6:**
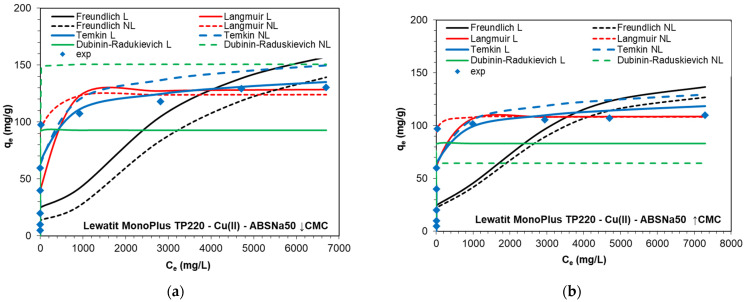
Fitting of the Langmuir, Freundlich, Temkin, and Dubinin–Radushkevich isotherms to experimental data using the linear (L) and nonlinear (NL) regression for the HCl-Cu(II) system with ABSNa50: (**a**) ↓CMC, *C*_0_ = 18 mg/L; (**b**) ↑CMC, *C*_0_ = 2550 mg/L) with Lewatit MonoPlus TP220.

As was found, the adsorption capacities of TP220 were equal to 130.38 and 110 mg/L for Cu(II) in the presence of ABSNa50 of the concentrations ↓CMC and ↑CMC, respectively. With the increasing concentration of the surfactant, a decrease in the adsorption capacity was observed. The isotherm data analysis using the Langmuir, Freundlich, Temkin, and Dubinin–Radushkevich (D–R) models (model descriptions in Section 3.4) was performed applying the L and NL regressions. The isotherm parameters are collected in Table 4, whereas the fitting of the calculated and experimental isotherms is depicted in Figure 6.

Based on the data obtained by applying the four isotherm models, it can be found that the highest *R*^2^ and *R^2^_adj_* values were obtained using the Langmuir isotherm model and the L and NL regressions. The *R*^2^ values were found to be 0.998 and 1.00 (L), as well as 0.962 and 0.978 (NL), while *R*^2^*_adj_* was equal to 0.951 and 0.972 (NL) for the system containing ABSNa50 ↓CMC and ↑CMC, respectively. Moreover, the monolayer sorption capacities (*Q*_0_) equal to 129.54 (↓CMC) and 109.08 mg/g (↑CMC) (L) and 124.06 (↓CMC) and 108.47 mg/g (↑CMC) (NL) are consistent with the maximum sorption capacity values obtained under the experimental conditions (*q_e_*_,*exp*_ = 130.38 (↓CMC) and 110 mg/g (↑CMC)). Relatively small values of *R*^2^ were obtained in the case of the Freundlich model (*R*^2^ = 0.711–0.718 (L) and *R*^2^ = 0.774–0.490 (NL). The 1/*n* parameter was in the range of 0.221-0.249 (L) and 0.224-0.313 (NL), and these values below 1 (1/*n* < 1) signify that the sorption of Cu(II) in the presence of ABSNa50 is favorable. The maximum adsorption capacities calculated from the D–R model (*q_m_* = 83.14–92.82 mg/g (L) and *q_m_* = 64.48–150.61 mg/g (NL)), as well as from the Temkin model (*q_m_* = 204.49–258.65 (L) and *q_m_* = 165.65–208.77 mg/g (NL)), do not match the experimental data. The D–R and Temkin models cannot be applicable for the description of these adsorption systems due to the fact that the obtained equilibrium data did not show a good fit to the model (*R*^2^ = 0.901–0.911 (L) and *R*^2^ = 0.851–0.953, *R*^2^*_adj_* = 0.809–0.940 (NL)—D–R; *R*^2^ = 0.890–0.922 (L) and *R*^2^ = 0.890–0.922, *R*^2^*_adj_* = 0.859-0.899 (NL)—Temkin). The estimated values of the mean free energy *E* from the D–R model could indicate the physical (*E* < 8 kJ/mol) or chemical nature (*E* = 8–16 kJ/mol) of adsorption, but the modeling of adsorption of Cu(II) in the presence of ABSNa50 on TP220 by the D–R model shows a small determination coefficient [57]. The adsorption capacities obtained during Cu(II) adsorption in the presence of the ABSNa50 surfactant on TP220 were compared with the other published ones concerning removal of Cu(II) by different adsorbents and are presented in Table 5. As was found, Cu(II) adsorption onto various adsorbents follows the Langmuir isotherm model. Moreover, the presence of the anionic surfactant ABSNa50 causes the decrease in the TP220 adsorption capacities towards copper (from 230.2 to 110–130.38 mg/g). On the other hand, the anion exchange resin still shows great adsorption ability towards copper.

### 2.4. Column Studies

The determination and description of the breakthrough curve (the volume of effluent collected to the breakthrough point, the time needed to reach the breakthrough point, and the shape of the breakthrough curve), as well as the column parameters calculation, can be a main feature of the fixed-bed column performance. The weight (*D_w_*) and bed (*D_b_*) distribution coefficients, as well as the working ion exchange capacity (*C_w_*), were calculated using the formulae [62]:

– the weight distribution coefficient (*D_w_*):
  (18) $$D_{w}=\frac{U^{\prime \prime }-U_{0}-V}{m_{j}}$$
  where *U*″(mL)—the eluate volume for *C/C*_0_ = 0.5, *U*_0_ (mL) —the dead column volume (*U*_0_ = 2 mL), *V* (mL)—the free volume (intergranular) of the ion exchange bed (about 0.4 bed volume), and *m_j_* (g)—the mass of dry ion exchange resin in the column;
– the bed distribution coefficient (*D_b_*):
  (19) $$D_{b}=D_{w}\times d_{z}$$
  where *d_z_* (g/mL)—the ion exchange density (determined experimentally by drying the appropriate amount of ion exchanger to the constant mass). The working ion exchange capacity (*C_w_*) (g/mL):
  (20) $$C_{w}=\frac{U_{p}\times C_{0}}{V_{j}}$$
  where *U_p_* (L)—the volume of eluate to break through the column, *C*_0_ (g/L)—initial concentration of Cu(II) in the solution, and *V_j_* (mL)—the volume of the ion exchange in the column.

**Figure 7:**
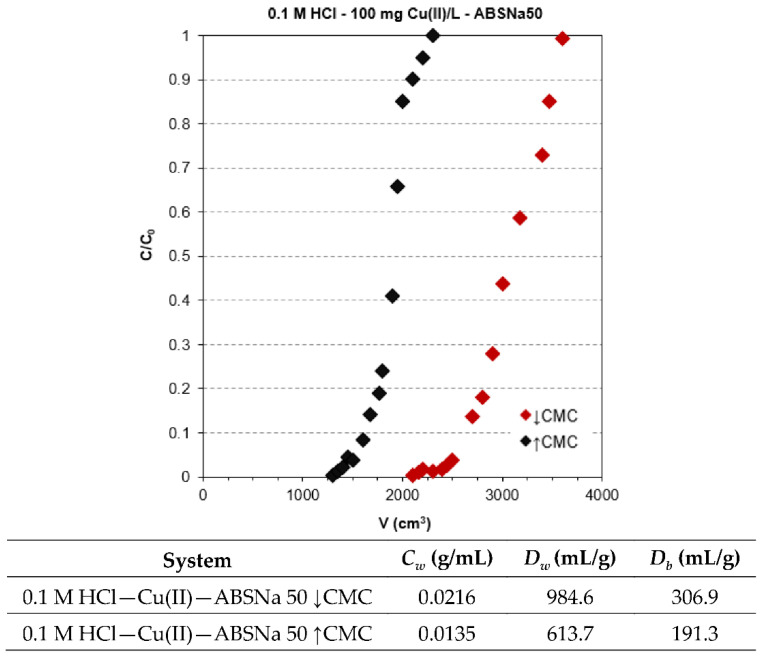
The breakthrough curves obtained during Cu(II) adsorption in the presence of ABSNa50 on TP220.

Cu(II) breakthrough curves in the presence of ABSNa50 on TP220 were determined and have a typical S-shape, as shown in Figure 7.

The addition of the surfactant at the concentrations ↑CMC and ↓CMC results in a decrease and increase in the volume of solution needed for the breakthrough point compared to the system without surfactant. The working ion exchange capacity is the highest for the 0.1 mol/L HCl–Cu(II)–ABSNa50 ↓CMC system (0.0216 g/mL), whereas for the system containing 0.1 M HCl–Cu(II) without the surfactant and 0.1 mol/L HCl–Cu(II)–ABSNa50 ↑CMC is equal to 0.0175 g/mL and 0.0135 g/mL, respectively. Additionally, the higher weight and bed distribution coefficients were found for the 0.1 M HCl–Cu(II)–ABSNa50 ↓CMC system (*D_w_* = 984.6 mL/g and *D_b_* = 306.9 mL/g).

### 2.5. Lewatit MonoPlus TP220 Characterization after the Cu(II) Adsorption in the Presence of ABSNa50 Surfactant

**Figure 8:**
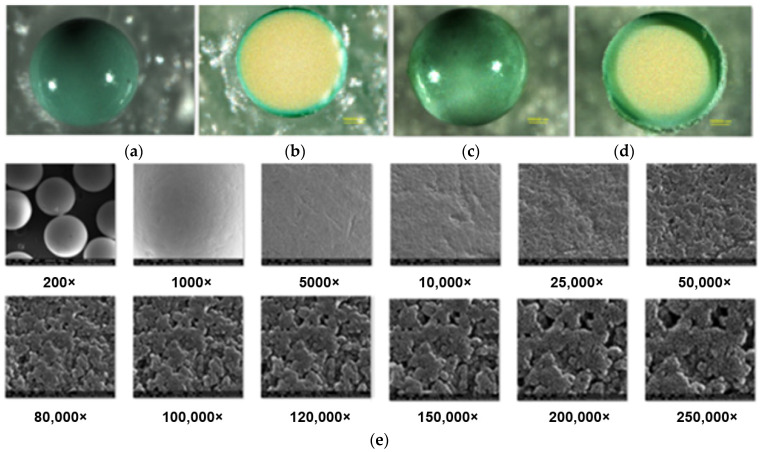
(**a**–**d**) Microscopic images (mag. 100,000×); (**e**) SEM images (mag. 200—250,000×) of Lewatit MonoPlus TP220 obtained: (**a**,**b**) after Cu(II) adsorption without and (**c**–**e**) with the ABSNa50 (↑CMC) surfactant from 0.1 mol/L solutions; (**a**,**c**)—the whole bead; (**b**,**d**)—the bead cut in the center.

After the Cu(II) adsorption in the presence of ABSNa50 process, the TP220 was analyzed using SEM, AFM, and an optical profiler to obtain additional information about the surface morphology, topography, and mechanism of adsorption (the SEM and AFM images of the TP220 before adsorption can be found in the previously published paper [22]. The SEM and AFM images (before adsorption) confirmed the monodispersity of the anion exchanger, the spherical shape of the spheres, and the rougher and highly porous structure [22]. Moreover, the topography of the sample and the comprehensive assessment of the microgeometry of the ion exchanger before adsorption show that the roughness of the surface (*R_a_*) of the unloaded ion exchanger is greater in the center than at the edge, where *R_a_* is the arithmetic mean of the elevation profile and one of the amplitude parameters applied for the characterization of the surface texture [23]. During Cu(II) adsorption, the color of the anion exchanger beads changes from cream/pale brown to green/blue (Figure 5 and Figure 8).

**Figure 9:**
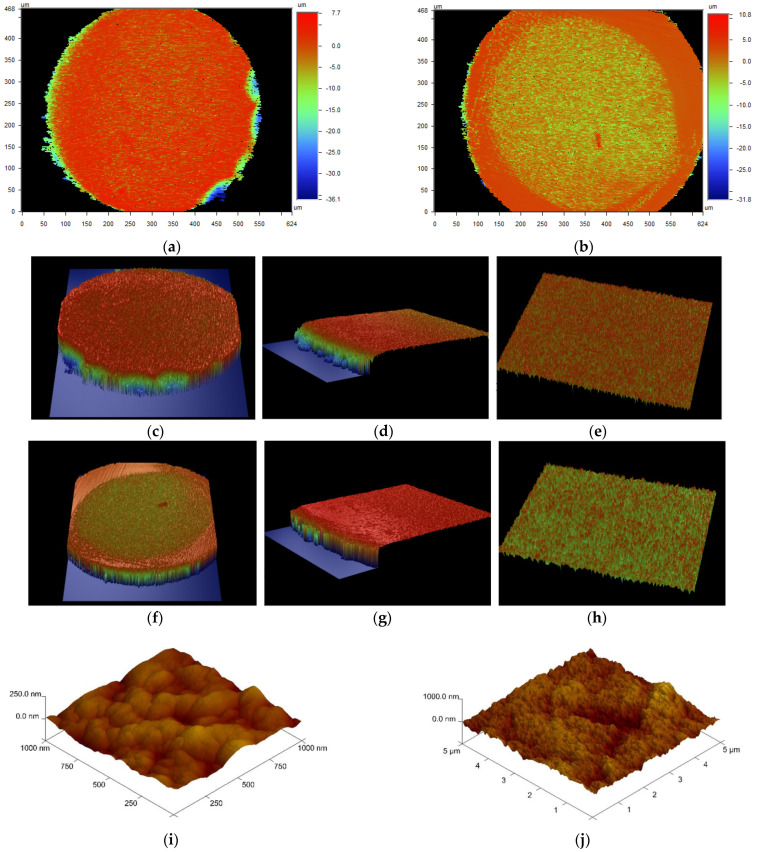
The images of Lewatit MonoPlus TP220 obtained from: (**a**–**h**) the optic profiler analysis and (**i**,**j**) AFM analysis after Cu(II) adsorption: (**a**,**c**–**e**) without and (**b**,**f**–**j**) in the presence of ABSNa50 (↑CMC) surfactant obtained from 0.1 M HCl solutions; (**a**,**b**)—2D, the whole bead; (**c**,**f**)—3D, the whole bead; (**d**,**g**)—3D, the edge; (**e**,**h**)—3D, the center; (**i**)—1 µm; (**j**)—5 µm.

Moreover, after copper adsorption in the presence of ABSNa50 on TP220, the *S_BET_* increases slightly from 21.38 to 28.26 m^2^/g (the average pore size is 46.7 nm; the total pore volume is 0.330 cm^3^/g). Furthermore, the increase in the *S_BET_* after Cu(II) adsorption without the surfactant on TP220 was previously observed (increase from 24.3 to 25.2 m^2^/g) [23]. Figure 8 presents the microscopic images of the whole or the cut-in-the-center bead of the ion exchanger after Cu(II) adsorption without the surfactant (a,b) and after Cu(II) adsorption in the presence of the ABSNa50 surfactant (c,d), while Figure 8e shows the structure of the surface (SEM images) obtained after Cu(II) adsorption in the presence of the ABSNa50 surfactant at different magnifications. As was found from the SEM images after Cu(II) adsorption in the presence of ABSNa50 (↑CMC), the shape of the beads did not change and the surface was still rough and possessed many pores and cavities. Based on the microscopic images (Figure 8a,c) of the whole bead after Cu(II) adsorption without the surfactant and in the presence of the surfactant, the adsorbent beads look similar, but looking at the microscopic images of the beads cut in the centre, the changes in the surface topography and adsorption layer thickness can be visually found. After Cu(II) adsorption without the surfactant, the adsorption layer thickness, visible as a ring in the cross-section of the bead, is small, whereas after Cu(II) adsorption in the presence of the surfactant, the adsorption layer thickness increases significantly. Such behavior was confirmed in the record three-dimensional surface views by the optical profiler (Contour GT) analysis. The images obtained during the optical profiler analysis of the ion exchanger bead after the Cu(II) adsorption without the surfactant and in the presence of the surfactant are presented in Figure 9a–h. It should be emphasized that this method is quick, non-contact, and allows the making microgeometry of the surface in a much greater measurement range (150 × 150 mm) than the AFM analysis (150 × 150 µm). Moreover, this method allows the analysis of materials with very large and very small roughness, as well as the whole beads of the ion exchangers. At the same time, it is worth emphasizing that this method is rarely used in the characterization of adsorbents, which is a novelty of the work. The AFM images of the ion exchangers after Cu(II) adsorption in the presence of the surfactant (↑CMC) are presented in Figure 9i,j.

The microgeometry analysis of TP220 by the optic profile confirmed that the roughness of the ion exchanger after the Cu(II) adsorption without or in the presence of the surfactant changed. The roughness of the surface (*R_a_*) after Cu(II) adsorption was *R_a_* = 2.63 µm for the whole bead, whereas *R_a_* was 0.760 µm for the edge and 0.842 µm for the center. After adsorption of Cu(II) in the presence of the ABSNa50ABSNa50 surfactant (↑CMC), the roughness of the surface was *R_a_* = 2.2 µm for the whole bead, whereas *R_a_* was 1.59 µm for the edge and 0.750 µm for the center. Such changes suggest that the adsorption of Cu(II) on TP220 proceeds at the edge of the beads (external adsorption) and also inside the beads (interial adsorption), whereas the adsorption of Cu(II) in the presence of ABSNa50 (↑CMC) is greater at the edge (the roughness increases significantly from 0.760 µm to 1.59 µm at the edge), and the thickness of the adsorption layer is much higher (average about 0.75 µm).

### 2.6. Copper(II) Removal by the Micellar-Enhanced Ultrafiltration (MEUF)

**Figure 10:**
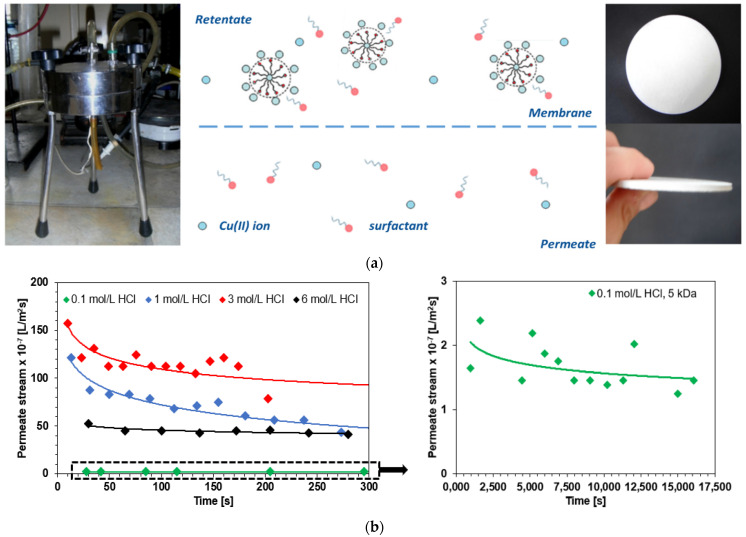

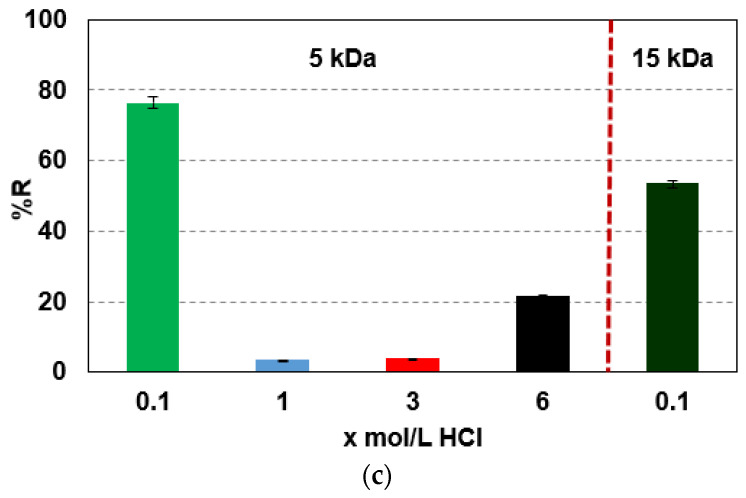
(**a**) Schematic illustration of the MEUF process including the apparatus and membrane used; (**b**) changes in the permeate flux over time during Cu(II) removal in the presence of the ABSNa50 (5 CMC, cut-off 5 kDa); (**c**) changes of degree of Cu(II) retention (%*R*) as a function of HCl concentration and membrane cut-off.

In addition, for comparison purposes, the micellar-enhanced ultrafiltration (MEUF) process was applied for Cu(II) removal (Figure 10a). The intensity (efficiency) of the membrane process is determined by the volume flux (*J_h_*) (L/m^2^∙h) or mass flux (kg/m^2^∙h) of the solution, the measure of which is the volume or mass permeating through the membrane under the driving force for a unit of membrane working area and a unit of time. The volume flux (*J_h_*) and the retention factor (degree of retention of the solute, %*R*) were calculated using the following formulae [63]:

(21) $$\%R=\left(1-\frac{C_{P}}{C_{R}}\right)*100\%$$

(22) $$J_{h}=\frac{V_{p}}{S\times t}$$

where *C_R_*—the initial concentration of the separated substance/ion (mg/L), *C_P_*—the concentration of the separated substance/ion in the permeate after time *t* (mg/L), *V_p_*—the volume of the permeate (L), *t*—the time to collect the sample, and *S*—the membrane effective area (m^2^).

Changes in the permeate flux over time during copper ion removal in the presence of the ABSNa50 anionic surfactant (5 CMC) as a function of hydrochloric acid concentration are presented in Figure 10b. During the process, the permeate flux decreases much more at the beginning of the filtration than at the end of the process. This is a consequence of the rapid development of concentration polarization on the membrane or fouling. The intensity of concentration polarization in the ultrafiltration was high, and it was caused by the retention of components on the membrane (more accumulation of solutes over the membrane surface took place during the extension of the operation time). The fouling based on the adsorption of particles inside the membrane pores results in the reduction of their inner diameter [64]. Here, the significant differences in the flux values obtained for 0.1 M HCl should be particularly emphasized. The observed, much higher resistance of the polarization layer on the one hand significantly limits the efficiency of the process (in the context of the obtained fluxes) but should contribute to the improvement of the efficiency of the process (in the context of the separation of metal ions). Additionally, the permeate flux depends on the used membrane. During the Cu(II) adsorption in the presence of ABSNa50 removal by the MEUF with a membrane characterized by a larger cut-off of 15 kDa (larger pores), a higher permeate flux is obtained, e.g., 8.02 × 10^−7^ L/m^2^s (15 kDa) and 1.64 × 10^−7^ L/m^2^s (5 kDa) for 0.1 M HCl and 960–1000 s, while its reduction with time is much larger than those for 5 kDa. The permeate flux reduction is equal to 11% and 21% for a membrane cut-off of 5 and 15 kDa, respectively. The degree of Cu(II) in the presence of ABSNa50 retention depends on the hydrochloric acid concentrations, as in Figure 10c. The addition of hydrochloric acid of higher concentrations results in a reduction in the Cu(II) ion (ABSNa50) retention (%*R* = 76.46%—0.1 mol/L HCl; 3.38%–21.54%—1–6 mol/L HCl, 5 kDa). Only at the HCl concentration of 0.1 mol/L is the ultrafiltration process effective with the retention above 76%, for a membrane with a cut-off of 5 kDa. Increasing the cut-off molar mass of the membrane decreases the retention rate by 23% (%*R* = 53.38%, 15 kDa). In spite of the fact that a ceramic membrane, known for its high resistance to high and low pH in contrast to the classically used polymeric membranes, was used in this study, the results show an unfavorable influence of the presence of hydrochloric acid on the efficiency of MEUF. The pH-dependent speciation of copper ions should also be taken into account. It can be assumed that in the pH range of 1–7 the divalent copper ions (Cu^2+^) and, in smaller amounts, the monovalent complexes predominate. The change of pH towards alkali (not present in this study) causes the formation of complexes, as well as the precipitation of copper hydroxide at pH 7–12 [K_sp_ Cu(OH)_2_ = 2.2 × 10^−20^] and the formation of anionic complexes Cu(OH)_3_^−^ at pH > 9 [65,66]. The pH of the solution therefore permits the presence of free, complexed, and molecular copper, and the above-mentioned aspects play a significant role in MEUF [67]. Such large changes in the degrees of yield for higher concentrations of acid compared to the 0.1 M system suggest a different behavior of the surfactant and its ability to solubilize metal ions, as well as their ionic form. The obtained results suggest that at low acid concentrations the interaction of copper ions with micelles is possible through electrostatic interactions (anionic surfactant-metal cation). On the other hand, as the acid concentration increases, a competitive reaction on the micelle surface may occur. Cu(II) ions bound to the micelles can be replaced by H^+^ ions present in the system. Both H^+^ and Cu^2+^ have a positive charge; so, H^+^ binds to the SDS micelles by electrostatic adsorption and occupies the binding sites, similarly to the copper ions. At low pH levels, the solution contains a large number of H^+^ molecules; so, the corresponding rejection of copper ions is relatively low. This confirms the previous studies on copper sorption with the mechanism shown in Figure 2, as well as the MEUF of metal ions at various pH levels [65,68,69]. Moreover, it should be noted that surfactant-metal ion and additional hydrogen ion interactions will be more important in terms of separation capacity than the membrane material itself. Regardless of the concentration of hydrochloric acid used, the zeta potential of the membrane is positive [70].

Table 6 presents Cu(II) removal by the MEUF method using different surfactants and membranes [10,37,65,66,67]. As was found, the copper removal by MEUF depends on the characteristics and concentrations of the surfactants, metals, solution pH, and parameters related to the membrane operation. Comparing the %*R* obtained during Cu(II) removal in the presence of ABSNa50 with the other ones, the degree of retention efficiency is similar for the other examples with similar membrane parameters, e.g., SDS/Triton X-100.

### 2.7. General Remarks

The obtained results were compared with the literature. Table 5 and Table 6 give such a comparison both for the adsorption and the MEUF methods. There were selected examples.

As was found in the literature, extensive research is being conducted to refine both treatment methods. Although all wastewater treatment techniques can be used to remove heavy metals, they have their inherent advantages and limitations, and the results presented here overcome some of the drawbacks. The removal of heavy metals from aqueous solutions, such as by chemical precipitation, is usually adapted to the treatment of heavy metal ion-containing wastewater with high concentrations of heavy metal ions and is ineffective when their concentration is low. The ion exchange method is widely used for the removal of heavy metal ions from wastewater. However, when the ion exchanger is exhausted, it must be regenerated with chemical reagents, which results in secondary contamination and can increase the total treatment technique cost, limiting its large-scale application [2]. Therefore, effective adsorbents that exhibit high contaminant-removal efficiency and have a high adsorption capacity are sought. Undoubtedly, the proposed TP220 exchanger meets the requirements in terms of pollutant-removal efficiency. The adsorption kinetics allows for the rapid removal of contaminants in a short time, which reduces costs, and the high ion-exchange capacity and the small reduction in capacity after successive sorption–desorption cycles also favorably affect the economics of the process. Moreover, the favorable results obtained provide an opportunity for the possible application of this ion exchange resin on a larger scale.

As for the MUEF method in literature, different uses of this method as a function of the compound to be removed and as a comparison of the different methodologies could be found. The recent patents and critical analysis of the literature data indicated that the most frequent gaps in MEUF studies are lack of information regarding the experimental parameters, such as pH, temperature, or permeate flux [67]. Therefore, the presented studies are very important from the cognitive point of view. Moreover, it was also observed that imprecise measurements of the CMC values could result in inaccurate or imprecise conclusions. Therefore, the main challenge for future industrial-scale applications of MEUF is to develop extensive studies of the MEUF wastewater-treatment method, including simple synthetic wastewater treatment at the laboratory scale to industrial wastewater at a pilot scale [67,76].

Despite the versatility of adsorption and MEUF as promising methods to remove different types of contaminants including, among others, heavy metal ions from water and wastewaters, there are future challenges still to overcome.

## 3. Materials and Methods

### 3.1. Reagents and Instruments

**Figure 11:**
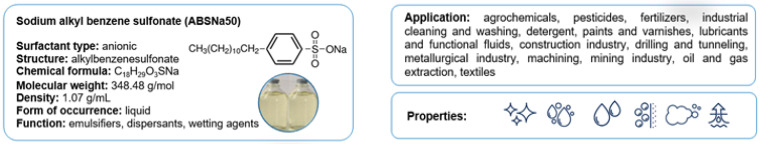
Sodium dodecylobenzene sulfonate (ABSNa50) characteristics.

Avantor and Fluka supplied all the essential chemicals of analytical grade. Copper(II) chloride, CuCl_2_·2H_2_O, and sodium dodecylobenzene sulfonate (ABSNa50) (Figure 11) of the chemical formula C_18_H_29_O_3_SNa and the molecular mass 348.48 g/mol, as well as hydrochloric acid (36–38% HCl), were applied for preparation of the working solutions (0.1–6 mol/L HCl–100 mg Cu(II)/L–ABSNa50 (18 mg/L (↓CMC) and 2550 mg/L (↑CMC)—estimation from the surface tension measurements). The atomic absorption spectrophotometer (AAS) (AA240FS Varian Inc, Belrose, Austalia) was used for examining the Cu(II) ion concentration in all samples.

### 3.2. Physical Characterization of Adsorbents

Eight sorbents, especially ion exchangers, were applied for Cu(II) removal from acidic solutions: Lewatit: AF5, MonoPlus TP220, or MonoPlus SR7, Purolite: A400TL, A830 or S984, and Dowex: PSR2 or PSR3. Their typical properties were taken from the product data sheets. Moreover, the sorbents were characterized by the Brunauer, Emmett, and Teller (BET) specific surface area and pore size, as well as by volume analysis. The Quantachrome ASiQwin instruments, version 3.01, USA (Brand of Anton Paar, Boynton Beach, Florida, USA)was applied to measure the adsorbent BET surface area, while the CHNS elementary analyses were conducted using Elemental Analyzer EA Vario EL III (Elementar, Hesse, Germany). The point of zero charge of the sorbents obtained by the solid addition method was also determined (0.5 g of the sorbent mechanically shaken with 50 mL of 0.1 mol/L KNO_3_ with different pH for 24 h). The point of zero charge, pH_pzc_, was determined from the intersection point between the difference in pH_f_ (final pH after 24 h) and the initial pH_0_ (before shaking) [30].

### 3.3. Sorption Studies by the Static and Column Tests

The static sorption of Cu(II) ions was tested in the conical flasks. First, 0.5 (±0.0005) g of a given adsorbent was weighed into flasks, and then, 50 mL of Cu(II) ions (100 mg/L) solution was poured. The prepared samples were placed in an Elpin+ mechanical shaker, type 357, with the following parameters: vibration amplitude, *A* = 8, shaking speed, V_as_ = 180 rpm, room temperature, and phase contact time, *t* = 1 min–24 h. Then, the aqueous phase was separated from the ion exchanger by filtration. Based on the obtained data, the sorption capacities (*q_e_*) and the amount of Cu(II) ions adsorbed on the given ion exchangers after time t (*q_t_*) (*q_e_* = *q_t_* for the phase contact time in which the system reaches equilibrium), as well as the percentage removal (%*R*), were determined.

The static method was also used to study the kinetics and equilibrium of the Cu(II) sorption process on the selected ion exchangers. The experimental conditions (the same: *m* = 0.5 g, *V* = 50 mL, amplitude: *A* = 8, shaking speed: *V_as_* = 180 rpm, room temperature, ABSNa50 concentration: 18 mg/L (↓CMC) and 2550 mg/L (↑CMC), and depending on the issue being examined, were as follows: (i) kinetic tests, influence of HCl and surfactant concentration: *C*_0_ = 100 mg Cu(II)/L, *t* = 1 min–24 h and (ii) equilibrium tests, determination of total adsorption capacity: *C*_0_ = 50–8000 mg Cu(II)/L, *t* = 24 h. For the selected ion exchanger (Lewatit MonoPlus TP220), the SEM, AFM, and optical profiler analyses for the samples after Cu(II) sorption in the presence of ABSNa50 were presented.

The dynamic method for Cu(II) sorption was tested in ion exchange columns with a diameter of *Ø* = 10 mm, connected to glass balloons by means of rubber hoses. The columns were filled with 10 mL of swollen Lewatit MonoPlus TP220 ion exchanger (selected based on the static studies). Then, the glass balloons were filled with Cu(II) solutions (0.1 M HCl–100 mg Cu(II)/L–ABSNa50 ↓CMC and ↑CMC) and the constant solution flow rate through the bed was kept at 0.4 mL/min. Leakage from the column—the eluate was collected in fractions with appropriate volumes until the initial concentration of Cu(II). The concentration of Cu(II) in the fractions was determined by the AAS method. Next, Cu(II) breakthrough curves were plotted for the tested systems (*C/C*_0_ dependence on *V* (mL), where *C/C* was the ratio of the concentration of Cu(II) in the eluate to that of Cu(II) in the solution introduced into the column), and the weight (*D_w_*) and volume (*D_b_*) distribution coefficients, as well as the working ion exchange capacity (*C_w_*), were calculated.

### 3.4. Modelling of Kinetic and Equilibrium Studies of Cu(II) Sorption

Adsorption and desorption are time-dependent processes. Therefore, knowledge of the rate of contaminant adsorption and desorption is important for the designing and evaluation of the adsorbent [77]. The pseudo-second order equation (PSO), the pseudo-first order kinetic equation (PFO), the Lagergren equation, and the intraparticle diffusion (IPD) kinetic models were applied for the adsorption system description (Equations (4)–(17)).

It was noted that ion exchangers, especially Lewatit MonoPlus TP220, are characterized by a significant sorption capacity, and Cu(II) has a high affinity to the surface of the examined adsorbent. The Langmuir [78], Freundlich [79], Temkin [80], and Dubinin–Radushkevich [51,81] isotherm models were used to study the equilibrium of Cu(II) sorption in the presence of ABSNa50 surfactant. The characteristics of the non-linear equation, along with the corresponding parameters, are presented in Table 7.

Based on the values of *R_L_* and 1/*n* parameters, the sorption process can be classified as favorable *(R_L_* = 0–1), unfavorable (*R_L_* > 1), irreversible (*R_L_* = 0), or when *R_L_* = 1, the linear isotherm can be obtained, whereas the parameter characterizing the energy heterogeneity of the adsorbent surface 1/*n* could be equal to 0—irreversible sorption, >1 unfavorable sorption or could be in the range from 0 to 1, then favorable sorption occurs [20].

All kinetic and equilibrium studies were carried out in three series, and the mean values of the experimental results (the standard deviation was in the range of 3–5%) were used for the data evaluation. Similar to the kinetic studies, the error analysis was also performed for the equilibrium studies, applying Equations (15)–(17).

### 3.5. Copper(II) Removal by the Micellar-Enhanced Ultrafiltration (MEUF)

The MEUF process, using a flat ceramic membrane (composite of titanium oxide with zirconia), was performed in the SPIRLAB cross-flow module by TAMI INDUSTRIES at 0.2 MPa, in which the circular cross-section membrane is located between two steel plates of the body cell. The filtration is realized in a tangential mode. The product circulates in a tangential manner on the disc membrane thanks to a superior spiral-shaped part. The permeate is recovered by an outlet situated underneath the disc-holder, and the retentate by an outlet is placed next to the inlet connection. The membranes (cut-off 5 ÷ 15 kDa) were used repeatedly and were conditioned in deionized water for 24 h before being used. For the separation of Cu(II) ions by MEUF, the solutions of copper(II) chloride salt with a concentration of 100 mg Cu(II)/L in 0.1, 1, 3 and 6 mol/L HCl were prepared with the addition of the anionic surfactant ABSNa50. Deionized water was passed through the installation to wash the system after each ultrafiltration.

## 4. Conclusions

In summary, the paper demonstrates the possibility of copper separation in the presence of surfactant, which is of considerable practical importance due to the presence of this type of fines in wastewater.

In this work, a series of ion exchangers was investigated for Cu(II) removal in the presence of the anionic surfactant ABSNa50 and hydrochloric acid, based on both static and dynamic methods. Moreover, a new approach to the determination of the adsorption mechanism was presented using the optical profiler method, which allows the obtaining of a detailed description of the topography and microgeometry of the ion exchanger beads after adsorption. It was shown that Lewatit MonoPlus TP220 is the most advantageous in the studied separation systems. The maximum adsorption capacities of TP220 towards Cu(II) in the presence of ABSNa50 were 110 mg/g (↑CMC) and 130.38 mg/g (↓CMC), and the kinetics followed the pseudo-second order kinetic equation. The working ion-exchange capacities were *C_w_* = 0.0216 (↓CMC) and 0.0135 g/mL (↑CMC). Based on the optical profiler method, it was concluded that the adsorption of Cu(II) in the presence of ABSNa50 (↑CMC) on TP220 occurs mainly at the edge of the beads. Additionally, the sorption method was compared with ultrafiltration. The adsorption method was found to give better Cu(II) removal efficiency (%*R* close to 100%), while the MEUF method had only %*R* = 76.46%. Moreover, the addition of HCl at higher concentrations and the increasing of the membrane molar mass cut-off resulted in a decrease in the retention of Cu(II) ions (ABSNa50) (from 76.46% to 53.38%, for 5 and 15 kDa; 0.1 mol/L HCl).

## Figures and Tables

**Table 1:** Characterization of ion exchange resins (based on the producer data sheets).

Sorbent	Type	Matrix	Structure	Functional Groups	Mean Bead Size (mm)	Total Capacity (val/L)	Water Retention (%)
AF5	AWFG	Carbonaceous	Micr.	without	0.4–0.8	–	48–60
S984	CHIE	Cross-linked polyacrylic	Macr.	Polyamine	–	2.7	44–55
TP220	CHIE	Cross-linked polystyrene	Macr.	Bis-picolylamine, bis(2-pyridyl-methyl)amine	0.62 (±0.05)	2.2	48–60
A830	WBA	Cross-linked polyacrylic	Macr.	Complex amine	0.3–1.2	2.75	47–53
A400TL	SBA	Cross-linked polyacrylic	Micr.	Quaternary ammonium, type 1	0.425–0.85	1.3	48–54
SR7	SBA	Cross-linked polystyrene	Macr.	Quaternary ammonium, type 3	0.57–0.67	0.6	59–64
PSR2	SBA	Cross-linked polystyrene	Micr.	Quaternary ammonium, tri-n-butyl amine type	0.3–1.2	0.65	40–48
PSR3	SBA	Cross-linked polystyrene	Macr.		0.3–1.2	0.6	50–65

WBA—weakly basic anion exchanger, SBA—strongly basic anion exchanger, CHIE—chelating ion exchanger, AWFG—adsorbent without functional groups, Micr.—microporous, Macr.—macroporous.

**Table 2:** CHNS characterization of ion exchange resins and carbon adsorbent.

Sorbent	%N	%C	%H	%S	*S*_BET_ (m^2^/g)	*P_s_* (nm)	*P_v_* (cm^3^/g)
TP220	9.26	70.58	7.81	0.24	21.38	48.20	0.258
SR7	2.58	70.79	6.49	0.26	19.69	44.61	0.220
AF5	0.02	93.81	1.58	0.91	988.77	2.31	0.572
A400TL	4.54	59.64	9.76	0.24	4.20	2.35	0.002
A830	16.35	46.00	8.39	0.00	9.66	48.83	0.012
S984	18.13	46.95	7.62	0.87	3.80	89.36	0.085
PSR2	2.49	71.38	8.02	1.28	6.32	10.08	0.016
PSR3	2.25	73.89	10.89	0.06	6.36	3.65	0.006

*P_s_*—average pore size, *P_v_*—total pore volume.

**Table 3:** Comparison of the kinetic parameters obtained for the HCl-Cu(II)-ABSNa50-TP220 systems.

Parameters		HCl−Cu(II)−ABSNa50	
		ABSNa50 ↑CMC	ABSNa50 ↓CMC
*q_e_*_,*exp*_ (mg/g)		9.97	9.99
PFO—L	*q_e,cal_* (mg/g)	2.39	1.08
	*k*_1_ (1/min)	0.006	0.003
	*R* ^2^	0.476	0.277
PFO—NL	*q_e_*_,*cal*_ (mg/g)	9.25	9.63
	*k*_1_ (1/min)	0.103	0.096
	*R* ^2^	0.975	0.989
	*R* ^2^ *adj*	0.969	0.986
	*MPSD*	0.115	0.026
PSO—L	*q_e_*_,*cal*_ (mg/g)	10.00	10.01
	*k*_2_ (g/mg min)	0.014	0.019
	*R* ^2^	1.000	1.000
	*h* (mg/g min)	1.44	1.89
PSO—NL	*q_e_*_,*cal*_ (mg/g)	10.16	10.61
	*k*_2_ (g/mg min)	0.012	0.010
	*MPSD*	0.027	0.027
	*R* ^2^	0.996	0.991
	*R* ^2^ *adj*	0.995	0.989
IPD	*q_e_*_,*cal*_ (mg/g)	14.47	14.57
	*k_i_* (mg/g min^0.5^)	0.18	0.18
	*R* ^2^	0.924	0.757
	*R* ^2^ *adj*	0.773	0.270

L—the linear regression, NL—the non-linear regression.

**Table 4:** List of calculated parameters obtained using the Langmuir, Freundlich, Temkin, and Dubinin–Radushkevich adsorption isotherm models.

Model	Parameters	HCl–Cu(II)–ABSNa50	
		ABSNa50 ↑CMC	ABSNa50 ↓CMC
	*q_e_*_,*exp*_ (mg/g)	130.38	110.00
**Linear regression**			
Langmuir	*Q*_0_ (mg/g)	129.54	109.08
	*k_L_* (L/mg)	0.021	0.044
	*R* ^2^	0.998	1.000
Freundlich	*k_F_* (mg^1−1/*n*^ L^1/*n*^/g)	19.20	21.04
	1/*n*	0.249	0.221
	*R* ^2^	0.718	0.711
Temkin	*b_T_* (J g/mol mg)	204.49	258.65
	*A* (L/mg)	10.494	32.460
	*R* ^2^	0.922	0.890
Dubinin–Radushkevich	*q_m_* (mg/g)	92.82	83.14
	*k_DR_* (mol^2^ J^2^)	3.6 × 10^−7^	1.6 × 10^−7^
	*E* (kJ/mol)	1.177	1.787
	*R* ^2^	0.911	0.901
**Non-linear regression**			
Langmuir	*Q*_0_ (mg/g)	124.06	108.47
	*k_L_* (L/mg)	0.137	0.298
	*MPSD*	0.523	0.285
	*R* ^2^	0.962	0.978
	$R_{adj}^{2}$	0.951	0.972
Freundlich	*k_F_* (mg^1−1/*n*^ L^1/*n*^/g)	9.89	19.01
	1/*n*	0.313	0.224
	*MPSD*	2.244	4.149
	*R* ^2^	0.790	0.774
	$R_{adj}^{2}$	0.731	0.709
Temkin	*b_T_* (J g/mol mg)	165.65	208.77
	*A* (L/mg)	3.297	7.570
	*MPSD*	0.488	0.534
	*R* ^2^	0.922	0.890
	$R_{adj}^{2}$	0.899	0.859
Dubinin–Radushkevich	*q_m_* (mg/g)	150.61	64.48
	*k_DR_* (mol^2^ J^2^)	9 × 10^−7^	1.5 × 10^−7^
	*E* (J/mol)	745.54	1827.56
	*MPSD*	2.910	1.488
	*R* ^2^	0.953	0.851
	$R_{adj}^{2}$	0.940	0.809

**Table 5:** The equilibrium parameters of Cu(II) sorption on various adsorbents.

Adsorbate/Adsorbent		Conditions	Isotherm Models Sorption Capacity	Ref.
Cu(II)	H_3_PO_4_-activated rubber wood sawdust *S_BET_* = 1673.86 m^2^/g	0.5 g; 100 mL; pH 6; initial; 20 mg/L	Langmuir, *q_max_* = 5.73 mg/g (303 K) *q_max_* = 5.70 mg/g (308 K) *q_max_* = 5.49 mg/g (313 K)	[52]
Cu(II)	watermelon rind	0.5 g; pH 5; 10 mg/L; 20 °C	Langmuir, *q_max_* = 6.28 mg/g	[58]
Cu(II)		1 g; pH 5; 10 mg/L; 40 °C	Langmuir, *q_max_* = 31.25 mg/g	[59]
Cu(II)	AC from hazelnut husks * S_BET_ = 1092 m^2^/g	0.05–0.5 g; 25 mL; 200 rpm	Langmuir, *q_max_* = 6.65 mg/g	[60]
**Ion exchangers**				
Cu(II)	Lewatit MonoPlus TP220 styrene divinylbenzene FG: bis-picolylamine	0.5 g; V = 50 mL; 100–10000 mg/L; 180 rpm; t = 24 h	Langmuir, *q_max_* = 230.2 mg/g	[30]
Cu(II)	Purolite S940, Purolite S950 styrene divinylbenzene FG: aminophosphonic	0.20 g; 20 mL of 0.001 mol/L CuCl_2_; 180 rpm; T = 298 K; T = 2 h	Based on the kinetic studies: *q* = 8.17 mg/g, S940 *q* = 9.72 mg/g, S950	[53]
Cu(II)	Purolite S930, ST-DVB FG: iminodiacetic acid	1 g/L; pH 3; 100, 300 mg/L; T = 293, 303 K; t = 1 min–24 h	Langmuir, *q_max_* = 133.33 mg/g	[54]
OS-20 + Cu(II) + H_2_O	Purolite C106 polyacrylic, FG: carboxyl groups	0.5 g; 100 mL; pH 3–5; 6 mmol/L; 20 °C	Based on the kinetic studies: *q* = 0.22 mmol/g, pH 3 *q* = 0.21 mmol/g, pH 5	[55]
ALM-10 + Cu(II) + H_2_O	Duolite ES 468 polyacrylic, FG: carboxyl groups	0.5 g; 100 mL; pH 3–5; 6 mmol/L; 20 °C	Based on the kinetic studies: *q* = 1.07 mmol/g, pH 3 *q* = 1.39 mmol/g, pH 5	[56]
ALM-10 + Cu(II) + H_2_O	Purolite C106 polyacrylic, FG: carboxyl groups	0.5 g; 100 mL; pH 3–5; 6 mmol/L; 20 °C	Based on the kinetic studies: *q* = 0.61 mmol/kg, pH 3 *q* = 0.46 mmol/kg, pH 5	[61]
Cu(II) + HCl + ABSNa50 ↓CMC	Lewatit MonoPlus TP220 styrene divinylbenzene FG: bis-picolylamine	0.5 g; V = 50 mL; 50–8000 mg/L; 180 rpm; t = 24 h	Langmuir, *q_max_* = 130.38 mg/g	This paper
Cu(II) + HCl + ABSNa50 ↑CMC		0.5 g; V = 50 mL; 50–8000 mg/L; 180 rpm, t = 24 h	Langmuir, *q_max_* = 110 mg/g	This paper

*q_max_*—the maximum sorption capacity, AC—activated carbon, *S_BET_*—the Brunauer–Emmett–Teller (BET) surface area, *—with ZnCl_2_ activation at 973 K in N_2_ atmosphere, FG—the functional groups, ALM-10—the non-ionic surfactant including alkylomonoethers, OS-20—the non-ionic surfactant oxyethylated alcohols.

**Table 6:** Copper removal by MEUF method.

Surfactant	Membrane	Pore Size	%R	Ref.
SDS/Triton X-100 10 mmol/L	cellulose, *S_a_* = 60 cm^2^	5 kDa	85%	[71]
SDS/Triton X-100 5.67/1.29 mmol/L	cellulose, YM10	10 kDa	92%	[72]
Brij 35:SDS 0.3 TW-80:SDS 0.3 Triton X-100:SDS 0.7	Polysulfone	6 kDa	98.3% 95.8% 93.5%	[37]
SDS	polyacrylonitrile *S_a_* = 550 cm^2^	5 kDa	98%	[73]
SDS/ligands *	Amicon hydrophilic YM10 membrane	10 kDa	>95% (without ligands); <40% (with ligands); <10% (SDS > 25 mmol/L)	[10]
CPC/ligands *			100% (EDTA: Cu = 1); 50% (EDTA: Cu = 24); 90% (NTA)	
RO90	cellulose PLCC	5 kDa	98%	[74]
SDBS	polysulfone membranes, *S_a_* ≈ 900 cm^2^	5 kDa	>90%	[75]
ABSNa50ABSNa50 (5CMC)	ceramic membrane ** S_a_ ≈ 64 cm^2^	5 kDa	76.46% (0.1 mol/L HCl)	This paper

*S_a_*—the total filter surface area, SDS—sodium dodecyl sulfphate, RO90—oleylethoxycarboxylate, CPC—etylpyridinium, * EDTA—ethylenediaminetetraacetic acid, NYA—nitrilotriacetic acid and citric acids, SDBS—sodium dodecylbenzenesulfonate, **—titanium oxide with zirconia.

**Table 7:** Characteristics of isotherm models.

Isotherm	Non-Linear Forms	Equation Number	Linear Forms	Equation Number
Langmuir	$q_{e}=\frac{k_{L}Q_{0}C_{e}}{1+C_{e}k_{L}}$	(23)	$\frac{C_{e}}{q_{e}}=\frac{1}{Q_{0}k_{L}}+\frac{C_{e}}{Q_{0}}$	(24)
Freundlich	$q_{e}=k_{F}C_{e}^{1/n}$	(25)	$log\;q_{e}=log\;k_{F}+\frac{1}{n}log\;C_{e}$	(26)
Temkin	$q_{e}=\frac{RT}{b_{T}}lnAC_{e}$	(27)	$q_{e}=\left(\frac{RT}{b_{T}}\right)lnA+\left(\frac{RT}{b_{T}}\right)lnC_{e}$	(28)
Dubinin– Radushkevich	$q_{e}=q_{m}e^{k_{DR}\varepsilon^{2}}$	(29)	$lnq_{e}=lnq_{m}-k_{DR}\varepsilon^{2}$	(32)
	$\varepsilon =RTln\left[1+\frac{1}{C_{e}}\right]$	(30)		
	$E=\frac{1}{\sqrt{2k_{DR}}}$	(31)		

where *q_e_* (mg/g)—the amount of Cu(II) ions adsorbed per unit mass of adsorbent, *C_e_* (mg/L)—the equilibrium concentration of solution, *Q*_0_ (mg/g)—the monolayer adsorption capacity, *k_L_* (L/mg)—the Langmuir constant (related to the free energy of adsorption), *k_F_* (mg^1-1/n^ L^1/n^/g) and 1/*n*—the Freundlich constants connected with adsorption capacity of adsorbent and the surface heterogeneity, *R* (8.314 J/mol K)—the gas constant, *T* (K)—the temperature, *A* (L/g) and *b_T_* (J/mol)—the Temkin constants, *q_m_* (mg/g)—the maximum adsorption capacity, *k_DR_* (mol^2^ J^2^)—the constant related to the adsorption energy, *ε* (J/mol)—adsorption potential, *E* (J/mol)—mean free energy for removing Cu(II) ions from its adsorption site to the infinity.

## Data Availability

All data used to support the findings of this study are included within the article.

## Sample Availability

Samples of the compounds are not available from the authors.
